# Effectiveness and safety of tralokinumab in a patient with severe atopic dermatitis and hepatitis B virus^؆^

**DOI:** 10.1016/j.abd.2025.501239

**Published:** 2025-11-01

**Authors:** María Olivares-Guerrero, Ana Jiménez-Sánchez, Mario Aparicio-Domínguez, Pablo Chicharro

**Affiliations:** Department of Dermatology, Hospital Universitario de La Princesa, Madrid, Spain

*Dear Editor,*

Atopic dermatitis (AD) is a chronic, inflammatory, and pruritic dermatological condition affecting millions of individuals worldwide.^1^ The treatment landscape for moderate-to-severe AD has significantly evolved in recent years with the advent of new biologic therapies, including tralokinumab. Tralokinumab is a monoclonal IgG4 antibody approved for the treatment of moderate-to-severe AD in adults and adolescents. It specifically binds to Interleukin-13 (IL-13), inhibiting its interaction with its receptors.^2^, ^3^, ^4^ Despite its efficacy, the emerging nature of these immunomodulatory therapies raises questions about their safety and effectiveness in populations with chronic infections, such as Hepatitis B Virus (HBV), as these patients are often excluded from clinical trials because of the risk of reactivation. While isolated case reports suggest that dupilumab may be safe in patients with chronic HBV, there are no specific data regarding tralokinumab.^5^ Here, we present the first documented case of a patient with moderate-to-severe AD and chronic HBV infection treated with tralokinumab.

A 77-year-old woman with a 30-year history of untreated chronic HBV infection presented to our dermatology department with intensely pruritic erythematous lesions over the past 3-years. The patient, originally from the Philippines, had a history of allergic rhinitis and flexural eczema during childhood. Since then, her eczema had remained reasonably well controlled, with occasional flares of pruritic lesions that she managed with topical corticosteroids prescribed by her general practitioner. Based on the diagnostic criteria of Hanifin and Rajka,^6^ she was diagnosed with AD. She had previously received up to 48 sessions of narrowband UVB phototherapy (cumulative dose: 43.567 mJ/cm^2^) and short-term courses of high-dose corticosteroids during disease flares, with partial response.

On physical examination, erythematous-brown plaques with a scaly surface were observed predominantly on the flanks and lateral aspects of the upper and lower extremities (Fig. 1A). The eczema area and severity index (EASI) was 31, and the body surface area affected was 21%. A skin biopsy was performed, revealing irregular acanthosis with spongiosis and occasional eosinophils. Direct immunofluorescence (DIF) of healthy skin was negative.

**Fig. 1:**
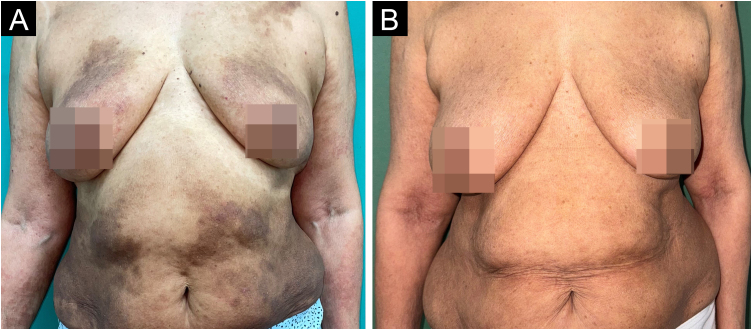
(A) Baseline clinical presentation prior to initiating tralokinumab. Erythematous-brown plaques in a phototype IV patient, symmetrically distributed primarily over the flanks, chest, and anterior thighs. (B) Clinical presentation after six months of tralokinumab. Attenuation of the lesions with blurring of the well-defined borders.

Initial laboratory evaluation revealed evidence of minimally replicative chronic HBV infection, with positive HBsAg, anti-HBc, and anti-HBe antibodies, but negative HBeAg and anti-HBs. The viral load was 1112 IU/mL. Serum lactate dehydrogenase (LDH) was elevated at 377 IU/L (reference range [RR] 135‒214 IU/L), and total IgE was >5000 KU/L (RR 0‒100), while other laboratory parameters, including liver enzymes, and anti-BP180, anti-BP230, anti-DG1, and anti-DG3 antibodies, were either negative or within normal limits. Peripheral blood immunophenotyping showed no abnormal cell populations. Tests for other hepatotropic viruses were negative, and abdominal ultrasonography ruled out hepatic lesions.

Given the lack of response to initial therapeutic approaches and the clinical progression of the disease, treatment intensification was deemed necessary. After consulting with the hepatology team, entecavir 0.5 mg/day was started to manage viral replication, and the use of immunosuppressive treatments such as cyclosporine was discouraged. Subsequently, tralokinumab therapy was started at a dose of 300 mg every two weeks.

Over the following months, the patient experienced significant clinical improvement, with a marked reduction in pruritus and lesions, leading to an EASI score of 3 by the sixth month of treatment (Fig. 1B). The therapy was well tolerated, with no adverse effects reported. Liver function remained stable, and HBV viral load decreased to <10 IU/mL during this period.

This is the first documented case of tralokinumab’s safety in a patient with active chronic HBV infection and moderate-to-severe AD. Previous reports have focused on dupilumab in similar contexts. Karen Ly and Mary Patricia Smith described two patients with AD and chronic HBV infection treated with concomitant dupilumab and entecavir.^7^ In one case, HBV viral load was already undetectable at the start of dupilumab treatment, while in the other, the viral load decreased from 19,120 IU/mL to undetectable before starting therapy. Matsutani and Imai conducted an observational study involving five patients with positive anti-HBc antibodies and no prior HBV vaccination who received dupilumab. Among these, only one patient exhibited transiently detectable HBV DNA at week-76 (<1.30 log IU/mL), which resolved spontaneously.^8^ However, all patients were HBsAg-negative at the initiation of treatment, and none had active or acute HBV infections.

Our case provides unique insights, as it involves a patient with untreated, active chronic HBV infection. The combination of entecavir and tralokinumab demonstrated safety, with no evidence of HBV exacerbation during treatment.

This case highlights the potential safety of tralokinumab in patients with active chronic HBV infection and AD. Although further studies are needed to validate these findings, this report underscores the importance of individualized approaches when managing patients with concurrent chronic infections and inflammatory skin diseases.

## ORCID ID

Mario Aparicio-Domínguez: 0009-0008-5646-3406

## Authors' contributions

María Olivares-Guerrero: Conceptualization; writing, and editing.

Ana Jiménez-Sánchez: Review; validation.

Mario Aparicio-Domínguez: Review; validation.

Pablo Chicharro: Supervision; review; validation; final review, and manuscript approval.

## Financial support

The research presented in this article has not received specific funding from public sector agencies, commercial entities, or non-profit organizations.

## Research data availability

Does not apply.

## Conflicts of interest

Pablo Chicharro has participated in advisory boards, symposia, and clinical trials organized by the following companies: Janssen Pharmaceuticals, Almirall, La Roche-Posay, Sanofi Genzyme, Lilly, AbbVie, Novartis, Leo Pharma, and Pfizer-Wyeth. The remaining authors declare no conflicts of interest.
